# Dietary and Flight Energetic Adaptations in a Salivary Gland Transcriptome of an Insectivorous Bat

**DOI:** 10.1371/journal.pone.0083512

**Published:** 2014-01-14

**Authors:** Carleton J. Phillips, Caleb D. Phillips, Jeremy Goecks, Enrique P. Lessa, Cibele G. Sotero-Caio, Bernard Tandler, Michael R. Gannon, Robert J. Baker

**Affiliations:** 1 Department of Biological Sciences, Texas Tech University, Lubbock, Texas, United States of America; 2 Department of Biology, Emory University, Atlanta, Georgia, United States of America; 3 Department of Math and Computer Science, Emory University, Atlanta, Georgia, United States of America; 4 Departamento de Ecología y Evolución, Facultad de Ciencias, Universidad de la República, Montevideo, Uruguay; 5 Department of Biological Sciences, School of Dental Medicine, Case Western Reserve University, Cleveland, Ohio, United States of America; 6 Department of Biology, Pennsylvania State University, Altoona College, Altoona, Pennsylvania, United States of America; Louisiana State University, United States of America

## Abstract

We hypothesized that evolution of salivary gland secretory proteome has been important in adaptation to insectivory, the most common dietary strategy among Chiroptera. A submandibular salivary gland (SMG) transcriptome was sequenced for the little brown bat, *Myotis lucifugus*. The likely secretory proteome of 23 genes included seven (*RETNLB*, *PSAP*, *CLU*, *APOE*, *LCN2*, *C3*, *CEL*) related to *M. lucifugus* insectivorous diet and metabolism. Six of the secretory proteins probably are endocrine, whereas one (*CEL*) most likely is exocrine. The encoded proteins are associated with lipid hydrolysis, regulation of lipid metabolism, lipid transport, and insulin resistance. They are capable of processing exogenous lipids for flight metabolism while foraging. Salivary carboxyl ester lipase (*CEL*) is thought to hydrolyze insect lipophorins, which probably are absorbed across the gastric mucosa during feeding. The other six proteins are predicted either to maintain these lipids at high blood concentrations or to facilitate transport and uptake by flight muscles. Expression of these seven genes and coordinated secretion from a single organ is novel to this insectivorous bat, and apparently has evolved through instances of gene duplication, gene recruitment, and nucleotide selection. Four of the recruited genes are single-copy in the *Myotis* genome, whereas three have undergone duplication(s) with two of these genes exhibiting evolutionary ‘bursts’ of duplication resulting in multiple paralogs. Evidence for episodic directional selection was found for six of seven genes, reinforcing the conclusion that the recruited genes have important roles in adaptation to insectivory and the metabolic demands of flight. Intragenic frequencies of mobile- element-like sequences differed from frequencies in the whole *M. lucifugus* genome. Differences among recruited genes imply separate evolutionary trajectories and that adaptation was not a single, coordinated event.

## Introduction

Among mammals, bats exhibit the greatest intraordinal diversification in diet. In general terms, diets range from insectivory and carnivory to frugivory and nectarivory and sanguivory [1]–[3]. Thus, pathways of bat adaptive radiation are defined by lineage differences in diet-associated enzymes, morphology (especially of the rostrum), dentition, salivary glands, and digestive tracts [4]–[10]. Flight is the centerpiece of bat biology. The constraints imposed by the morphological and physiological requirements of flight are interrelated to the diverse chiropteran dietary adaptations. Flight is metabolically the most expensive form of locomotion [11], [12]. Moreover, recent physiological studies have shown that insectivorous bats use exogenous lipids to fuel their flight within minutes of onset of feeding [13]. How this feat is achieved, is unknown although it is obvious that either the digestive process is accelerated or that alternative metabolic pathways are involved.

Salivary glands evolve rapidly, are involved in dietary adaptations, and release both exocrine and endocrine products [4], [7], [14]–[16]. We hypothesized that the submandibular salivary glands (also known in literature as ‘submaxillary’ glands) have had a direct role in bat adaptation to insectivory and possibly an indirect role in flight, given the rapid processing of exogenous lipids required for refueling flight muscles while feeding. The evolution of this capability could involve recruitment and expression of genes encoding secretory proteins with lipid-processing functions. If salivary glands had an important role in adaptive radiation in mammals, as has been proposed, it probably is due to the fact that additions and deletions to a salivary gland secretory proteome in a particular species could occur quickly and regularly [15]. Salivary glands have been described as a test bed for new, adaptive, roles for secretory proteins [15].

In addition to evidence that the secretory proteome is not conserved, there also are generic differences in salivary gland phenotypic characters (cell ultrastructure, histology, and histochemistry) that correlate with phylogenetic topologies and can be diet-associated [4]. For example, lysozyme-c, which is hypothesized to function both as a pH-dependent chitinase and as an antibacterial enzyme, is produced by different cells and in different salivary glands in various species of bats. Patterns such as these are compatible with the hypothesis that salivary glands offer multiple opportunities for gene recruitment and expression [14]. In terms of expression sites in particular salivary gland secretory cells, orthologous genes have had independent evolutionary trajectories in different bat lineages [14]. Thus, in some insectivorous species lysozyme-c-like immunoreactivity is associated only with acinar cells, whereas in other species in different bat families expression is associated with intercalated duct cells [14].

**Figure 1:**
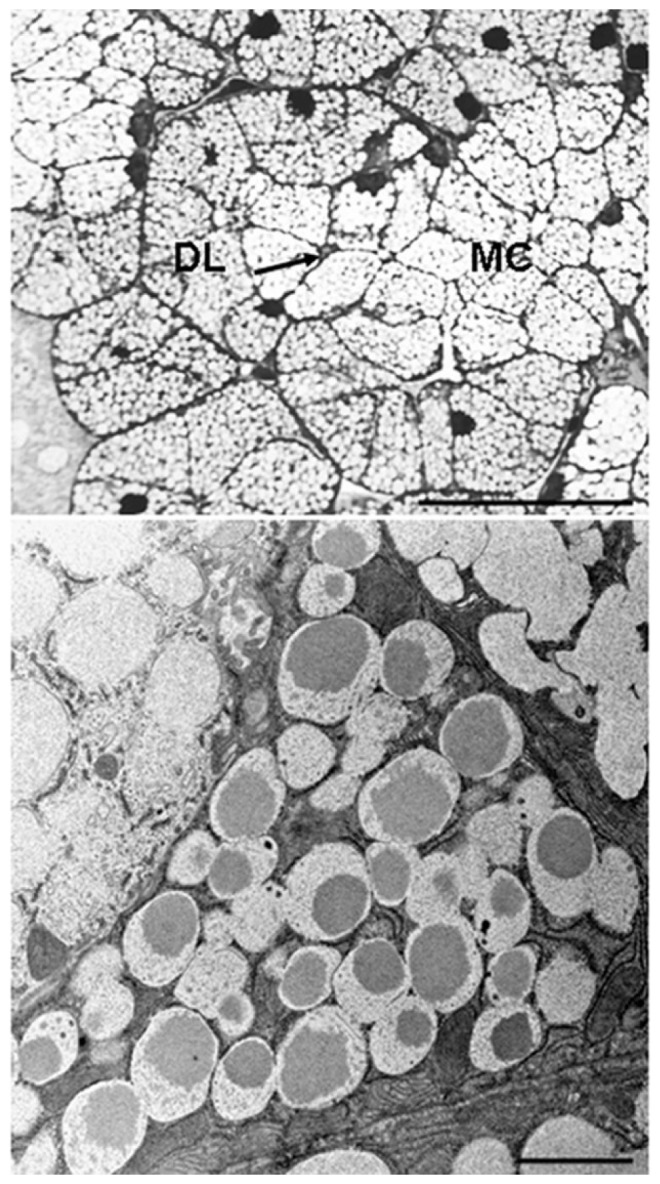
Optical micrograph of a semithin section of a secretory endpiece in a *Myotis lucifugus* principal submandibular gland, (A). The more central mucous cells (MC) are surrounded by slightly darker demilunar seromucous cells (DL). The endpiece lumen is indicated by the arrow. Toluidine blue. Scale bar = 40 µm. B. Transmission electron micrograph of a demilunar seromucous cell flanked by mucous cells. Note the difference in the structure of the secretory granules in the respective cell types. Scale bar = 2 µm.

We think that insectivory was the original microbat diet and that the evolution of flight, echolocation, digestive tract, excretory system, and metabolic physiology all are related to exploiting lipid-rich insects as the primary source of energy. To test our hypothesis about the role of the submandibular salivary gland, we sequenced the transcriptome from the principal submandibular salivary gland (SMG) of the little brown bat, *Myotis lucifugus*. We then used this transcriptome data set to identify a putative secretory proteome for this gland in this species. The large, paired, principal submandibular salivary glands in *Myotis lucifugus* are positioned medial to the angular process of the mandible. The gland has a conserved histological structure and the secretory endpieces, intercalated ducts, and striated ducts all are involved in regulated secretion [17]. Although histological structure is preserved, the secretory endpieces in the *Myotis lucifugus* SMG are unusual in comparison to bats in other families because they consist of mucous tubules capped by seromucous demilunes (Fig. 1A). Transmission electron microscopy reveals a variety of secretory granules in the cell cytoplasm and differences in granule size and morphology among cell types (Fig. 1B). Ultrastructural diversity in secretory granule contents within a particular SMG is a consequence of physiochemical differences among secretory products [4], [18].

*Myotis lucifugus* is an insectivorous bat. It feeds on soft-bodied insects, especially moths, captured in flight or by gleaning from vegetation [19]. Nutritionally, moths are a rich source of lipids. A specialized fat body accumulates and stores lipids in the pupa. The stored lipids are the energy source for metamorphosis and the fat body in the adult provides energy for flight and reproduction [20]–[22]. The genetic and physiological adaptations that have enabled insectivorous bats to use this insect resource for fueling flight and basic metabolism are currently unknown. Specifically, we selected *M. lucifugus* because: 1) the histology and mucous histochemistry of its SMG have been analyzed [17]; 2) there are many unresolved metabolic and energetic questions; and because 3) the *M. lucifugus* genome is available (www.ensembl.org) as a reference point for genetic analyses.

Transcriptomes of selected organs or tissues are an effective way of comparing gene expression, discovering interspecies differences, and identifying putative intracellular and secretory proteins. This is particularly the case in glands that have both exocrine and endocrine secretory functions. Gene expression in such glands is dynamic and interspecific differences are commonplace [23]. In our study, we surveyed the SMG transcriptome for genes that encode secretory proteins known to be associated in mammals with lipid hydrolysis, transport, hyperlipidemia, and energy metabolism. Expression of a cluster of such genes in the SMG would support our hypothesis and would illuminate specific adaptations to a lipid-rich insectivorous diet. Potentially, such results would also help explain the remarkably rapid use of exogenous lipids during foraging flights.

## Results and Discussion

### Seven Key Genes in the Secretory Proteome

The intact SMG consists of a duct system with four epithelial cell types known in *Myotis lucifugus* to exhibit regulated secretion (demilune, acinar, intercalated, and striated duct cells). In addition to data for these cell types, the SMG transcriptome sequences included products of genes expressed in myoepithelial and endothelial cells, fibroblasts, cells in blood plasma, and a substantial number and variety (in terms of types) of neurons.

To construct a putative secretory proteome for the salivary gland itself, we focused on expressed genes encoding products known to be associated with a) regulated secretory cells (rather than constitutive secretory cells) such as acinar or pancreatic beta cells or intestinal epithelium or b) extracellular (post-secretion) lipid metabolism. A set of 23 genes met criterion ‘a’ and thus constitute our putative secretory proteome; seven of these 23 genes in the *M. lucifugus* SMG transcriptome fit both ‘a’ and ‘b’ criteria (Table 1). Co-expression of these seven genes in the secretory proteome of *M. lucifugus* SMG is known only from *Myotis* and thus is unique among mammals for which we have comparative SMG data either from literature or Expressed Sequence Tags (EST). We propose that these seven genes have had adaptive roles in terms of salivary gland evolution, lipid metabolism, and, in general terms, the insectivorous diet and metabolism of *M. lucifugus*. Expression levels of these seven genes varied from a FPKM value (fragments per kilobase of exon model per million fragments mapped) of 355.6 to 30.2 as calculated using Cufflinks [24].

**Table 1:** Summary for seven genes recruited to the secretory proteome of the submandibular salivary gland in the little brown bat, *Myotis lucifugus*.

Gene	Protein	FPKM value (mRNA)	Pathway	Typical Expression Sites	Lipid-associated Function(s)
*RETNLB*	Resistin-like β	355.6	Endocrine	Intestinal epithelium (restricted)	Lipidemia and insulin resistance
*LCN2*	Lipocalin 2	353.4	Endocrine	Liver and adipose cells	Lipid transport
*C3*	Complement 3	191.4	Endocrine	Liver and adipose cells	Lipidemia, lipid transport
*PSAP*	Prosaposin	139.9	Endocrine	Kidney tubules, mammary glands	Lipid transport
*CLU*	Clusterin	60.9	Endocrine	Intestine, pancreas, liver	Lipid transport
*APOE*	Apolipoprotein E	35.1	Endocrine	Liver, adipose cells, macrophages	Lipid transport
*CEL*	Carboxyl ester lipase	30.2	Exocrine	Pancreas, lactating mammary gland	Lipid hydrolysis

The predicted secretory pathway (exocrine or endocrine) is based on protein function. Expression sites selected as typical are based on a combination of literature and EST profile.

Four of the seven secretory protein genes listed in Table 1 are single-copy genes in the *Myotis* genome. The other three genes (*LCN2*, *C3*, *CEL*) have undergone duplications. In each case only one of the duplicated paralogous genes is expressed in the SMG. Expression sites for the other *LCN2*, *C3*, and *CEL* paralogs in *Myotis* are presently unknown. All seven of the genes vary substantially in terms of their ‘typical’ tissue expression sites across mammals. We used UniGene (www.ncbi.nlm.nih.gov/UniGene), Expressed Sequence Tag (EST) profiles, and literature mining to determine approximate expression patterns for the seven genes of interest. EST profiles are available for ‘salivary glands’ (presumably the submandibular or parotid gland, or both) of humans, mice, cow, and swine. From the profiles we know that the seven genes of interest are not expressed as a set in salivary glands of these four species.

### Single-copy Genes

#### Resistin-like B protein (RETNLB; ENSMLUG00000001459)

Mammals typically have a single *RETNLB* gene. In *Myotis lucifugus* the gene spans 2.2 kb, is structured in three exons separated by two introns, and encodes a 115-amino acid cysteine-rich hormone. Analysis of intron 1 in the *M. lucifugus RETNLB* gene revealed a SINE-like sequence, whereas intron 2 contains a DNA/hAT-like sequence. Under the REV model, codon 7 in the N-terminal of the predicted *M. lucifugus* RETNLB protein is under positive selection. The EST profile for the *RETNLB* gene in mammals indicates typical expression only in intestinal epithelium (especially colon). There has been debate about the multiple functions attributed to RETNLB protein. The subject is complicated because although the *RETNLB* gene typically is expressed in intestinal epithelium, the *RETN* gene (which encodes resistin) is expressed in adipose tissue [25], [26]. Expression of the *RETNLB* gene in *M. lucifugus* SMG is unique and further complicates understanding of functional similarities and differences and roles of resistin-like B and resistin. It is relevant to our assessment of adaptation in insectivorous bats that circulating resistin-like B hormone has been shown to increase insulin resistance and to be associated with hyperlipidemia [26]. It also has been shown to regulate lipid energy metabolism and homeostasis [27]. This hormone probably is an endocrine product of *M. lucifugus* SMG (Table 1).

#### Prosaposin (PSAP; ENSMLUG00000015746)

Mammals typically have a single *PSAP* gene. In *M. lucifugus*, the gene spans 15.4 kb, is structured in 14 exons separated by 13 introns, and encodes a 523 amino acid precursor protein. Mobile element-like sequences occur in introns 1–3 and 5–8, but are absent from intron 4 and introns 9–13. The REV model did not detect any statistical evidence of prosaposin codons under episodic directional selection in *M. lucifugus*. The predicted prosaposin protein in *M. lucifugus* is 87% identical to the ortholog in the fruit bat, *Pteropus alecto*. Data are not currently available for comparison with other bat species. The EST profile indicates nearly ubiquitous *PSAP* gene expression in humans, laboratory mice, and swine. The gene is expressed at very low levels in human and mouse salivary glands, but not in swine salivary glands. The PSAP protein is thought to be secreted before its final processing and the secreted form serves as a lipid transporter that delivers bound sphingolipids to cell plasma membranes and into an endocytotic pathway [28]. The intracellular prosaposin peptides —referred to as saposins (termed A, B, C, and D)—enhance lysosomal hydrolytic activity [29].

#### Clusterin (CLU; ENSMLUG00000006721)

Mammals typically have a single *CLU* gene. The *M. lucifugus CLU* gene spans 12.6 kb, is structured in eight exons separated by seven introns, and encodes a 449 amino acid protein. Mobile element-like sequences occur in introns 1–7. The predicted *CLU* protein is 95% identical to the ortholog in congeneric *M. davidii* and 78% identical to the *Pteropus alecto* ortholog. The REV model identified one codon (238) as under episodic directional selection in the *Myotis lucifugus CLU* gene. The EST profile for *CLU* estimates that it is expressed principally in brain, joints, lymph, and pituitary glands, but also appears in numerous other tissues including liver. The clusterin protein has both intra- and extracellular functions [30], [31]. Numerous functions have been ascribed to clusterin, including service as a chaperone protein [31]. In blood, the CLU protein binds with and transports high-density lipoprotein (HDL) particles to the megalin cell surface receptor [32].

#### Apolipoprotein E (APOE; ENSMLUG00000006546)

Mammals typically have a single *APOE* gene. In *M. lucifugus*, the gene spans 3.3 kb, is structured in three exons separated by two introns, and encodes a 310 amino acid protein. No mobile element-like sequences were found in the intragenic introns of the *M. lucifugus APOE* gene. The REV model identified episodic directional selection in the ancestral gene tree branch leading to both *Myotis* and a fruit bat *Pteropus vampyrum* for which a genome database is available. The predicted APOE protein in *M. lucifugus* shares 87% identity with the orthologous protein in congeneric *M. davidii*, and only 70% with the orthologous protein in the vampire bat, *Desmodus rotundus*. The EST profile documents *APOE* expression principally in liver and adipose cells, but the gene is widely expressed at lower levels. The secreted form of *APOE* consists of two domains—the NH2-terminal domain that binds to the low-density lipoprotein (LDL) cell surface receptor and the COOH-terminal that binds to LDLs [33]. In humans, three *APOE* alleles encode proteins that are associated with differing lipoprotein plasma concentrations [34]. The APOE protein probably is secreted as an endocrine product from *M. lucifugus* SMG (Table 1).

### Multiple-copy (duplicated) Genes

#### Lipocalin 2 (LCN2; ENSMLUG00000016210)

Duplications of the *LCN2* gene have occurred independently in several mammalian lineages (particularly rodents and carnivores). In *M. lucifugus*, a single duplication has produced two *LCN2* genes (ENSMLUG00000016210 expressed in SMG and ENSMLUG00000029693 with unknown expression site(s)). The *LCN2* gene expressed in the SMG spans 3.4 kb, is structured in six exons separated by five introns, and encodes a 199 amino acid protein. No mobile element-like sequences were identified in the introns. Gene 1 (ENSMLUG00000016210) is the most conserved paralog of the two *LCN2* genes in *M. lucifugus*. Gene 1 shares 85% identity with the orthologous gene in *M. davidii* and 80% identity with the *Pteropus alecto* ortholog. The REV model estimates that the gene tree branch separating the duplicated *LCN2* genes in *M. lucifugus* from the single copy *LCN2* gene in *Pteropus vampyrum* has been under episodic directional selection. The EST profile for *LCN2* indicates that the gene is expressed, albeit at low levels, in a variety of tissues and cells. In humans, the highest expression level is in bone marrow but it also is expressed in liver and adipose tissue.

Lipocalin 2 is a cytokine with a role in regulating lipid metabolism and increasing insulin resistance [35], [36]. The protein has the β-barrel motif similar to other lipocalins, including an array of secretory and intracellular lipid-binding proteins [37], [38]. In the extracellular milieu, the LCN2 protein can bind to fatty acids or iron and has been investigated from a health perspective in connection with obesity, insulin resistance, and inflammation [39], [40]. It also modulates the peroxisome proliferator-activated receptor-γ (PPAR- γ), which in turn regulates energy expenditure and lipid homeostasis in adipocytes [41]. The *Myotis lucifugus* SMG protein isoform most likely is secreted as an endocrine product (Table 1).

#### Complement component 3 (C3; ENSMLUG00000011254)

**Figure 2:**
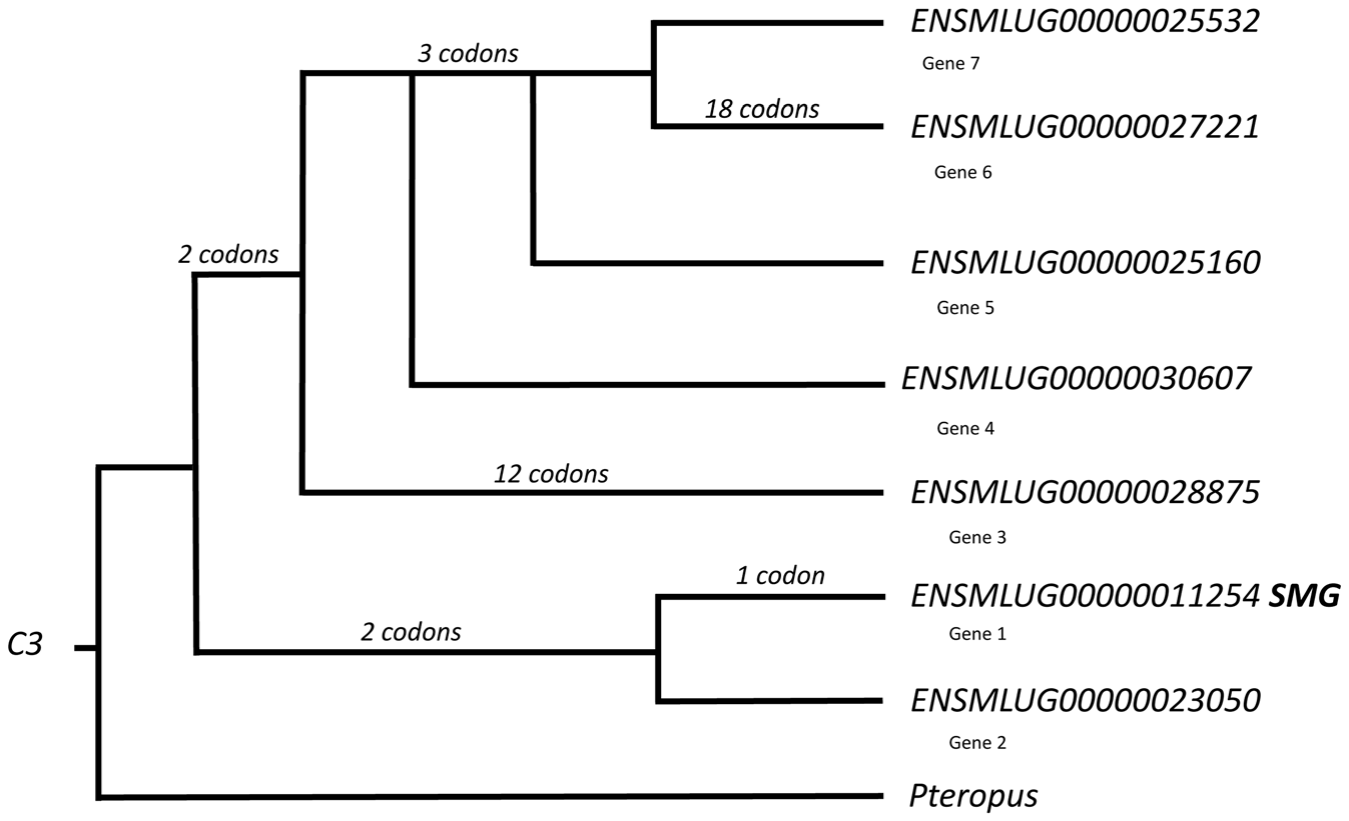
Gene tree for the *C3* genes in *Myotis lucifugus*. Numbers of codons under episodic positive selection are shown for each lineage where they occur. Gene ENSMLUG00000011254 is expressed in the principal submandibular gland (labeled SMG). Genes are numbered G1-G7 to correspond to Figure 5 and text.

In most (35/40 genera) mammals for which we have genome databases, *C3* is a single copy gene. *Myotis lucifugus* is one of 5 exceptions to the rule; in the genome of this bat, six gene duplications account for seven paralogous *C3* genes (Fig. 2). The fruit bat, *Pteropus vampyrum*, has only a single copy of the *C3* gene in its genome. The *C3* gene expressed in the *M. lucifugus* SMG (ENSMLUG00000011254, Gene 1) spans 32.2 kb, is structured in 42 exons separated by 41 introns, and encodes a large, 1664 amino acid glycoprotein. The size and structure of Gene 1 and primary structure of the encoded protein in *Myotis lucifugus* are conserved relative to orthologous *C3* genes in other mammals. The predicted *M. lucifugus* Gene 1 protein is 78% identical to the single ortholog in *Pteropus alecto* (data unavailable for *M. davidii*). Mobile element-like sequences and simple repeat motifs occur in 14 of 41 introns (34%) in *Myotis C3* Gene 1. The REV model identified episodic directional selection in Gene 1. In mammals with a single copy of the *C3* gene, EST profiles indicate expression level is highest in liver, but that *C3* also can be expressed at low levels in a variety of other tissues. Low level expression is seen in mouse salivary gland, but not in human or swine submandibular glands.

The mammalian *C3* gene encodes a complex precursor molecule [42]. Cell-specific processing of the large precursor protein results in one of two potential peptides that are cleaved from the precursor protein [43]–[45]. In hepatocytes, enzymatic cleavage within the N-terminal of the nascent C3 protein produces a 77 amino acid anaphylatoxin peptide (C3a) with immunological functions [43], [46]. The C3 protein that remains after cleavage is termed C3b. Consequently, hepatocytes secrete two proteins—the anaphylatoxin peptide (C3a) and a large C3b protein—both of which are processed from a single precursor. An alternative processing occurs in adipose cells. In these cells the C-terminal arginine is cleaved from the anaphylatoxin peptide. This modification of the anaphylatoxin peptide creates a functionally different peptide known as acylation-stimulating protein (ASP; C3adesArg) [47]–[49]. Removal of the terminal arginine eliminates any of the immunological properties associated with anaphylatoxin peptide. Instead, ASP is associated with triacylglycerol clearance from the blood [50]. In summary, in humans, laboratory mice, and rats, adipose cells secrete ASP and C3b and hepatocytes secrete anaphylatoxin peptide (C3a) and the C3b (complement component 3) protein.

The cell-specific ASP pathway in adipose cells requires co-expression of two other genes, complement factor B (*CFB*) and complement factor D (*CFD*), that encode cytoplasmic (non-secretory) proteins involved in the processing of the *C3* gene secretory product [48]. With regard to expression of a *C3* gene in *M. lucifugus* SMG, we asked whether the salivary gland uses the adipose cell ASP pathway or the alternate anaphylatoxin (C3a–C3b) processing pathway typically seen in hepatocytes. Based on the transcriptome data, the *CFB* gene (ENSMLUG00000000609) is expressed in the *Myotis* SMG, whereas the *CFD* gene is not expressed in the *M. lucifugus* submandibular gland (although it is present in the genome). Thus, we hypothesize that the *M. lucifugus* SMG secretes the anaphylatoxin peptide, C3a, as in liver rather than ASP as in adipose cells. In terms of known functions, anaphylatoxin peptide is immunological, the alternative protein, ASP, is associated with triacylglycerol clearance, and the C3b protein is associated with insulin resistance and FFA (free fatty acid) trapping. Over-production of the C3b protein has been linked to hyperlipidemia in humans [51]. If the same is true for *Myotis*, then hyperlipidemia resulting from the abundant secretion of the C3b protein would be advantageous in processing and using insect lipids while foraging. The C3 protein probably is an endocrine secretory product from *M. lucifugus* SMG (Table 1).

#### Carboxyl ester lipase (CEL; ENSMLUG00000006710)

**Figure 3:**
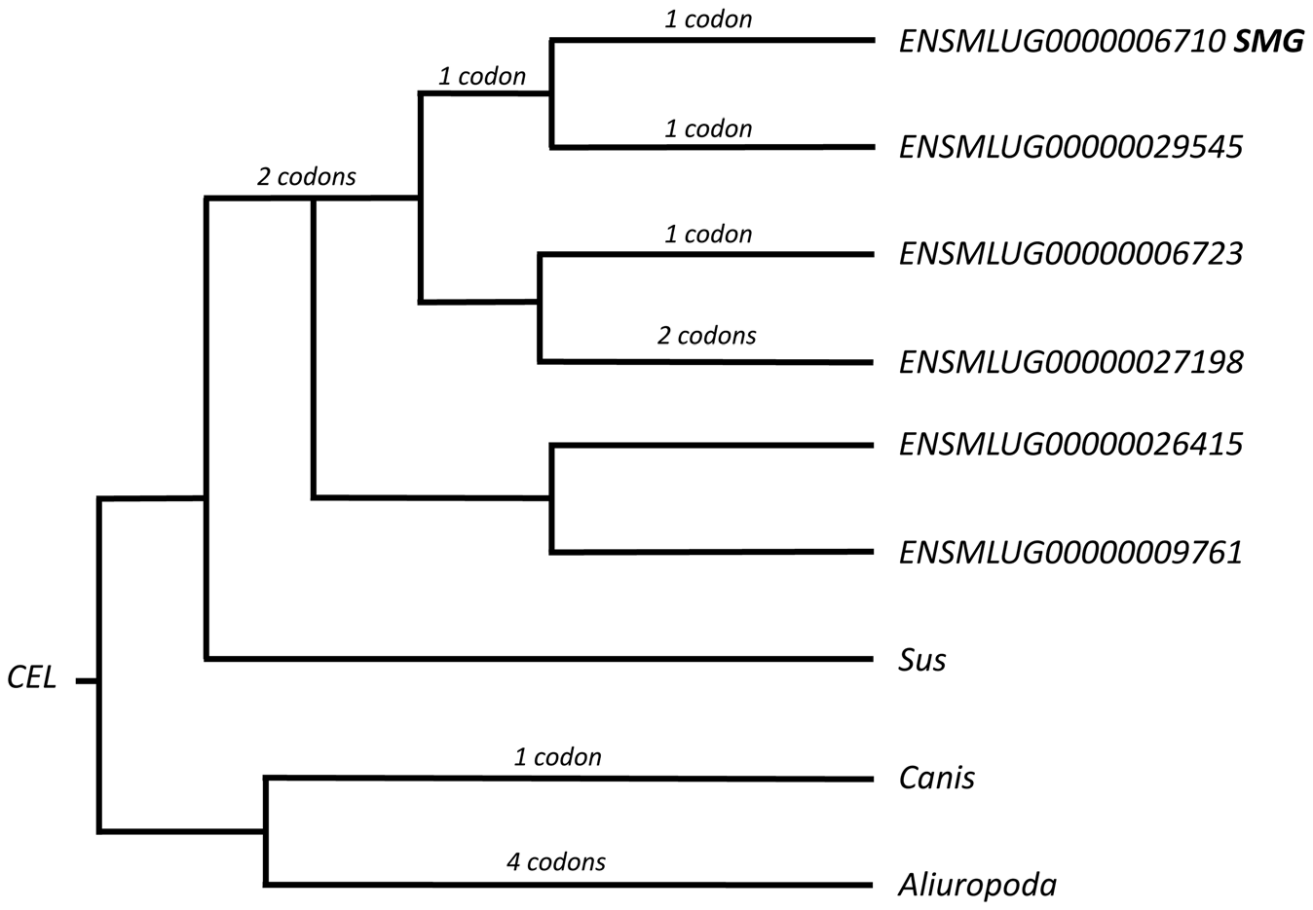
Gene tree for the *CEL* genes in *Myotis lucifugus*. Numbers of codons under episodic positive selection are shown for each lineage where they occur. Gene ENSMLUG00000006710 is expressed in the principal submandibular gland (labeled SMG).

Mammals typically have a single *CEL* gene. *Myotis lucifugus* is 1 of 2 species (out of 33 for which genome data are available) that exhibits *CEL* gene duplications. The fruit bat, *Pteropus vampyrum*, has a single copy of the *CEL* gene. In the *M. lucifugus* genome, five hypothesized gene duplications have produced a total of six paralogous *CEL* genes, one of which (ENSMLUG00000006710, labeled Gene 1; Fig. 3) is expressed in the SMG. Gene 1 spans 18.4 kb and is structured in 11 exons separated by 10 introns. Expression in the *M. lucifugus* SMG is predicted to produce two transcripts (ENSMLUT00000006710 and ENSMLUT00000022459) that result in protein isoforms of 573 and 560 amino acids, respectively. In comparison to *M. davidii*, which also has 6 paralogous *CEL* genes [52], Gene 1 in *M. lucifugus* shares 82–91% identity. Gene 1 is only 76% identical to the *CEL* gene in *Pteropus alecto*.

Multiple mobile element-like sequences occur in introns in Gene 1. The REV model identified episodic directional selection in this gene (Fig. 3). The EST profiles show that in mammals a *CEL* gene typically is expressed in pancreatic acinar cells. Expression also has been reported in lactating mammary glands of humans and laboratory rodents and in rat SMG striated ducts [53]. Striated duct cells frequently are secretory in the SMG and diet-associated patterns do occur [7]. Data from rats and mice and broad interspecific comparisons illustrate some of the difficulty in comparing possible expression across species in absence of transcriptome data [7]. Functionally, *CEL* encodes a bile-salt activated pancreatic acinar cell enzyme that hydrolyzes lipids and is associated with their intestinal absorption [54], [55]. It also has a role in neonate lipid metabolism and gastric absorption [56]. Presumably, the isoforms of carboxyl ester lipase secreted by the *Myotis* SMG are exocrine and thus are components of saliva in this bat.

### Evolutionary Mechanisms

Seven genes with interrelated lipid-associated functions are expressed in the *M. lucifugus* submandibular salivary gland transcriptome. The expression of these seven genes in a salivary gland secretory proteome is unique to *M. lucifugus* (among mammals for which we have comparable data) and probably is the result of multiple evolutionary mechanisms. We presently have no equivalent transcriptome-based secretory proteome data for any other insectivorous bat, or bats with any other diet for that matter. Consequently, we have no basis for estimating the evolutionary history of the SMG secretory proteome found in *Myotis*. A testable hypothesis based on the current research is that events resulting in expression of a group of seven genes with lipid-associated secretory products occurred early in the evolution of microbats, probably predating the origin of vespertilionids and certainly predating the origin of the genus *Myotis*.

The term ‘recruitment’ is suitable for describing the acquisition of gene expression in a novel cellular or tissue location. We assume that the seven genes of interest were recruited by various means for SMG expression early in microbat evolution. Although recruitment of gene expression is an important mechanism of adaptation, basic questions about the process are unanswered. For example, is recruitment of gene expression stochastic? Does the opportunity for novel salivary gland expression await some event such as insertion of an ERV/LTR transposon? Amylase expression in human salivary glands is an example of transposon involvement [57], [58]. But Cabej [59] has argued that under certain circumstances gene recruitment could be directly controlled by innervation, which is consistent with the role of innervation in salivary gland gene regulation and secretion [60]. Although acquisition of seven genes in the *Myotis* SMG proteome was important, it is unknown whether it was a coordinated process or the consequence of multiple independent stochastic events. In our opinion, the observation that recruited genes have different histories of duplication and expression profiles across tissues supports the argument that the *Myotis* SMG proteome evolved through multiple complementary, if not concerted, events rather than unrelated (independent) stochastic events.

#### Single Copy Gene Recruitment to the SMG

Among the single copy genes, recruitment of *RETNLB* expression to *Myotis* SMG perhaps is the most interesting example. Typically, expression of the RETNLB gene is regarded as specific to intestine [25]. Expression recruitment of single-copy genes, as opposed to duplicates, likely conserves protein primary sequence. RETNLB is a peptide hormone and recruitment of the gene for SMG expression presumably conserves the peptide's interactions with receptors and its function(s) as well. But what would be the adaptive advantage of recruiting a gene whose secretory product normally appears in the circulation anyway? Endocrine secretion from the *Myotis* SMG would place resistin-like B hormone directly into the circulation independent of the intestinal pathway to the liver. Its normal secretion is triggered by lipids in the intestine and in fact *RETNLB* gene expression is up-regulated by a high fat diet [27]. Secretion from the SMG would link release into the circulation with feeding rather than being dependent on lipids reaching the intestine. Such an explanation is consistent with a rapid response to the availability of exogenous lipids.

#### Gene Duplications

Gene duplications are an essential mechanism of genetic adaptation. Zhang et al. [52] reported that gene duplications appear to have played unusually important roles, perhaps including speciation, in *Myotis*. Gene duplications are believed to provide opportunities for structural and functional diversity in proteins insofar as paralogs can follow independent evolutionary trajectories [61], [62]. The *C3* and *CEL* genes in *Myotis* exhibit multiple paralogs requiring 5–6 duplication events. Their gene trees portray ‘evolutionary bursts’ because of the large number of paralogs produced. Closer examination of each of the paralogs revealed that gene truncations are common and that some of the paralogous genes have premature stop codons probably rendering them nonfunctional (Figs. 2, 3). Among the *Myotis C3* genes, three of the seven paralogs (Genes 1, 3, 6; Fig. 2) are structurally-conserved full-length genes, three of the genes (Genes 4, 5, 7; Fig. 2) are truncated and have early stop-codons, and one paralog (Gene 2; Fig. 2) is truncated so that it encodes a conserved version of the C3b protein without a premature stop codon. This latter gene presumably is functional. Among the three full-length *Myotis C3* paralogous genes, the codons under episodic directional selection (P>0.05; EBF>20) are asymmetrically distributed in terms of gene tree topology. Most of these codons are found in two lineages, one leading to Gene 6 (ENSMLUG00000027221) and the other leading to Gene 3 (ENSMLUG 00000028875). Our conclusion that three of the seven *C3* paralogs in *Myotis* have diverged under positive selection is consistent with the expectation for gene duplication [62]. An evolutionary burst and divergence of three paralogous *C3* genes in *Myotis* might be indicative of the important biological roles for the proteins encoded by this gene. It also is consistent with the conclusion that the *C3* gene and its encoded secretory product are an important part of the history of dietary and metabolic adaptation in insectivorous bats.

The carboxyl ester lipase gene (*CEL*) in *Myotis* also exhibits an evolutionary burst. In this instance gene duplications have resulted in six paralogs (Fig. 3). One to two codons under episodic directional selection (P>0.05; EBF>20) were identified in four of the six gene lineages (Fig. 3). Unlike the case of the *C3* gene, it does not appear that *CEL* paralogs are rapidly diverging under positive selection.

#### Novel Regulation for a Recruited Gene

The *NR5A2* (previously *LRH-1*) gene has been shown to regulate carboxyl ester lipase (*CEL*) expression in pancreatic acinar cells [55]. Gene duplication and recruitment of expression to the *M. lucifugus* SMG did not include the pancreatic regulatory gene, *NR5A2*. The absence of *NR5A2* from the salivary gland transcriptome indicates *CEL* gene expression in the SMG is regulated differently than is *CEL* gene expression in pancreatic acinar cells. So far, at least, it is typical that genes recruited to salivary gland secretory proteomes arrive without their regulatory systems. Rennin and amylase are just two examples from human and rodent salivary glands [63], [64]. The consequence is that recruitment is more than just novel gene expression and protein secretion from the salivary gland. The data suggest that *de novo* regulation of a recruited gene adds another opportunity for evolutionary diversification.

#### Genomic locations of SMG-expressed paralogs

**Figure 4:**
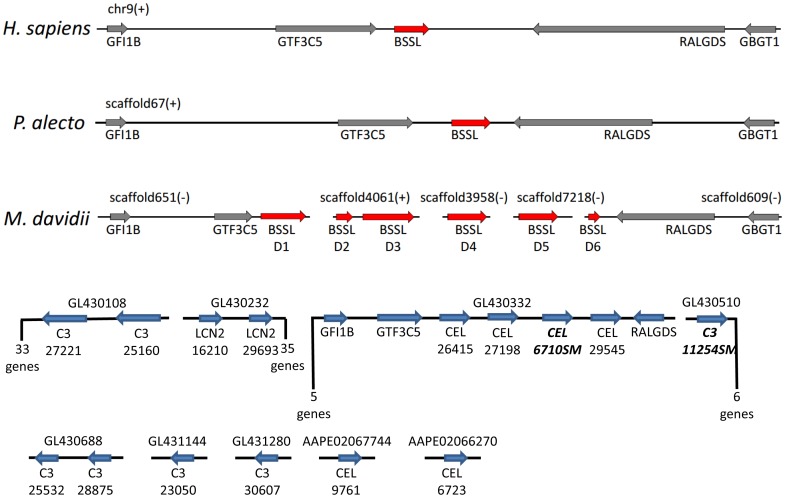
Comparison of relative genomic locations and gene order based on shared scaffolds in *Myotis lucifugus* (bottom in blue) with similar data from *M. davidii*, *Pteropus alecto*, and human beings. The comparative data are from Zhang et al. [52], who used the symbol ‘BSSL’ for the carboxyl ester lipase (*CEL*) genes.

Although we do not yet know the chromosomal location(s) of genes expressed in the *Myotis lucifugus* SMG, we used the order of assembled contigs in the Ensembl genomic database to explore relative positions of some of the secretory protein genes expressed in *M. lucifugus* SMG. The analogous region in humans is on chromosome 9 (9q34-34.3), which has a cluster that includes single copies of *LCN2* and *CEL* along with *GTF3C5*, *RALGDS*, and *GBGT1*. Zhang et al. [52] partially aligned their *M. davidii* data with the human genome and the fruit bat, *Pteropus alecto*, and showed the location of six copies of the *CEL* gene in this *Myotis* species (Fig. 4). Currently for *M. davidii*, the six *CEL* paralogs are assembled into five scaffolds, two of which include *GTF3C5* and *RALGDS*, but spatial relationships among scaffolds are unknown [56]. The Ensembl scaffolding for the *M. lucifugus* genome places four of six *CEL* paralogs on a scaffold with *GTF3C5* and *RALGDS* (Fig. 4). The other two *CEL* genes are on separate scaffolds. These comparisons suggest genomic rearrangements among the *CEL* gene cluster within the genus *Myotis* (Fig. 4). Additional investigations of patterns of congeneric chromosomal rearrangements are warranted because such studies will shed light on evolutionary rate and because bat chromosomal rearrangements already have been explored in substantial detail. Evidence points to rearrangements as important aspects of evolution and speciation [65], [66].

#### Mobile elements

Genomic databases and bioinformatic algorithms provide substantial new insights in the possible role(s) of mobile elements in shaping the mammalian genome [67]–[69]. Mobile elements (ME) had an important role in genome evolution specifically in *Myotis* and some categories of transposons possibly are still active in these bats [70], [71]. This particularly is the case with DNA/hAT elements, which are abundantly represented in *Myotis* [70]. In terms of salivary glands, for more than 20 years it has been known that a combination of gene duplications and transposable elements (especially LTR-retrotransposons) are associated with the recruitment of genes encoding secretory proteins [63], [64], [72], [73].

With the foregoing in mind, we determined the identities of ME-like sequences occurring in introns within each of five genes (*PSAP, CLU, RETNLB, C3, CEL*) containing such sequences. For comparison, we used the published data on the percentages of Class 1 and 2 MEs identified in the entire *Myotis* genome [74]. In making such a comparison it is essential to remember that our data are intragenic, whereas Pagán et al. [74] analyzed the entire genome, including intergenic sequence.

The set of *C3* paralogous genes consists of three full-length and four truncated genes. Moreover, two of the full-length genes have 12–18 codons under episodic directional selection. In comparison to the overall genome-wide distribution of MEs summarized by Pagán et al. [74], the intragenic distribution of ME-like sequences in the *C3* paralogs differed significantly (P>0.05) in three ways: 1) percentage-wise the *C3* paralogs had more intragenic Class 1 LINE (1 and 2) elements than the genome (24.17vs 17.45±4.27%); 2) more intragenic DNA/hAT element-like sequences (19.43vs 12.55±3.95%); and 3) more intragenic DNA/Tigger-like sequences (7.11vs 0.27±2.56%)(95%CI = *p*±1.96(*p*(1-*p*)/*N*)^0.5^). Additionally, the frequency of helitrons in the *Myotis* genome is 16.25%, whereas helitron-like sequences were absent from intragenic introns in the *C3* paralogs. Given the diversity and evolutionary history of the *Myotis C3* paralogs (truncated vs full-length; functional vs non-functional, positive episodic selection vs no statistical evidence of selection), we also asked if there were significant differences among ME frequencies in these genes. But no statistical difference was found (*Χ* ^2^ = 47.5; d.f. = 40; *P*>0.2). Overall, then, the relative intragenic percentages of various Class 1 and 2 mobile elements among paralogous *C3* genes is conserved.

The carboxyl ester lipase (*CEL*) genes provide us with a second example. In this instance the six paralogs differ remarkably from each other; one gene (Gene 1, expressed in the *Myotis* SMG) exhibits multiple intronic ME-like sequences, but all five of its paralogs have only 2–5 intronic MEs. MER20 (DNA-hAT/Charlie) sequences are the exception. In *M. lucifugus*, a MER20 sequence is conserved in all four of the functional *CEL* paralogs. MER20 has been associated with recruitment and creation of novel regulatory pathways [75]. In contrast, no MER20 sequences were found in the *C3* paralogs. The differences in the relative frequencies of Class 1 and 2 intragenic MEs in the duplicated *CEL* and *C3* genes in *M. lucifugus* is evidence of independent evolutionary histories for these genes, both of which are expressed in the SMG.

Likewise, differences among the single copy genes (*PSAP, CLU, RETNLB*) document that they also have had independent evolutionary histories (Table 2; Fig. 4).

**Table 2:** Summary of frequency (given in percentages) of mobile-element-like sequences in the *Myotis lucifugus* genome (from Pagán et al. ref 74) compared to intragenic frequencies (given in percentages) in the introns of four genes recruited to the submandibular salivary gland proteome.

	Genome	*C3*	*CEL*	*CLU*	*PSAP*
*Class1*					
SINES	39.86	41.23	42.86	31.58	41.6
LINES	17.49	24.17	0	10.53	16.6
ERV/LTR	9.7	8.06	23.81	15.79	8.33
*Class2*					
DNA/hAT	12.55	19.43	14.29	42.11	33.3
DNA/Tigger	0.27	7.11	0	0	8.33
Helitrons	16.25	0	14.29	0	0
Mariner	3.32	0	4.76	0	0
PiggyBac	0.65	0	0	0	0

**Figure 5:**
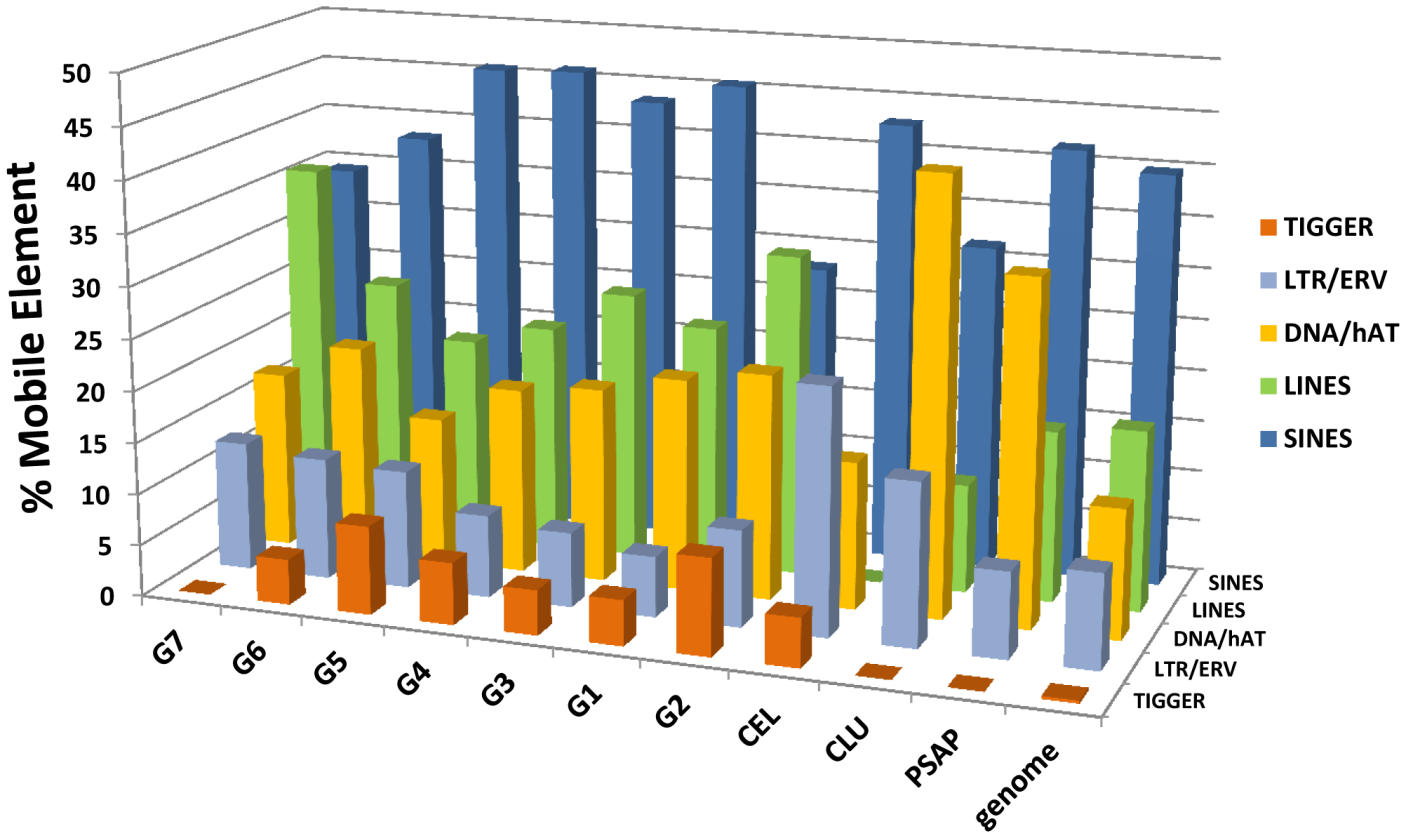
Histogram comparing percentages of Class 1 and 2 mobile element-like sequences in intragenic introns of selected *Myotis lucifugus* genes. The relative percentages of mobile element-like sequences in the total genome are shown on the right (from Pagán et al. [74]). Seven paralogs of C3 are shown, labeled G1–G7 (see Figure 2). See text for a full discussion of the data.

The data for *C3* are for all 7 paralogs. The data for *CEL* are from Gene 1 (based on 21 mobile element sequences in this gene), which is expressed in the SMG. The 5 paralogous *CEL* genes have only 2–5 mobile sequences/gene. See Fig. 5 for graphical presentation.

No single pattern of difference in ME distribution emerged from our comparisons among five recruited genes (including two that exhibited evolutionary bursts of duplication). At the same time, all five differed dramatically from the respective percentages of Class 1 and 2 mobile elements in whole genomic dataset. In the case of *C3*, the differences could be tested and were found to be statistically significant. The SINE-like sequences provided the only conserved pattern (Fig. 4). In the whole genome dataset, SINES comprise 39.86% of the elements and in all paralogs of *C3* and *CEL* and in *PSAP* and *CLU*, SINES range from 31.58 to 42.86% of the elements (Table 2). One striking difference between our intragenic data and the genome overall is the low frequency of LINES (1 and 2) sequences in two of the genes, *CEL* (0) and *CLU* (10.53), compared to the genome overall (17.45). DNA/Tigger-like sequences were prominent in introns in these genes, both of which had undergone an evolutionary burst (*C3, CEL*). Helitron-like sequences made up 14.29% of the ME-like sequences in the *CEL* gene recruited and expressed in *Myotis lucifugus* SMG (Table 2; Fig. 5). One relatively recent and demonstrably active DNA/transposon (*piggyBat*) known from the *M. lucifugus* genome [71], [76], was not detected in our intragenic dataset.

In summary, the occurrence of significant differences between intragenic and whole genomic distributions of certain MEs is evidence that MEs are not randomly positioned within the genome. Whether or not MEs had a role in recruitment of expression to the *Myotis lucifugus* SMG is unknown, although the data do support the hypothesis that all of these seven genes of interest have had independent evolutionary histories.

### Function and Proposed Adaptive Role(s)

Bats depend on flight for obtaining nutrients, but flight is metabolically expensive. The fact that bats typically spend nearly all of their non-foraging time in energy-saving physiological torpor with lowered body temperature and respiration shows that they live in a delicate metabolic balance. Bat flight muscles rely on fat as the oxidative substrate, whereas the capacity for glycogen metabolism is low or essentially nonexistent [77]–[80]. The emphasis on fat in bat metabolism has been attributed to its high density of energy storage (8× more density efficient) as compared to glycogen [13]. It also is the case that stored glycogen is heavier than stored fat because of the water co-located with glycogen. This in turn raises the physiological question of water balance in bats, which might limit glycogen storage. Moreover, exogenous rather than stored lipids are used to sustain foraging flight in insectivorous bats within minutes of consuming insects [13]. In a typical mammal, exogenous nutrients of all types are processed through a slow postprandial digestive pathway. Fats, for example, are hydrolyzed in the intestine, absorbed by enterocytes, and often converted to chylomicrons in a multistep intracellular process [81]. In fatty diets, chylomicron formation also inhibits gastric emptying through a feedback loop involving cholecystokinin [82]. Chylomicrons are exported from the enterocytes and carried away from the intestine via the lymphatic system. Usually the next steps are circulatory system clearance and storage in adipose cells or liver. In contrast, stable carbon isotope ratios have been used in insectivorous bats to show the rapid assimilation of exogenous lipids [13].

**Figure 6:**
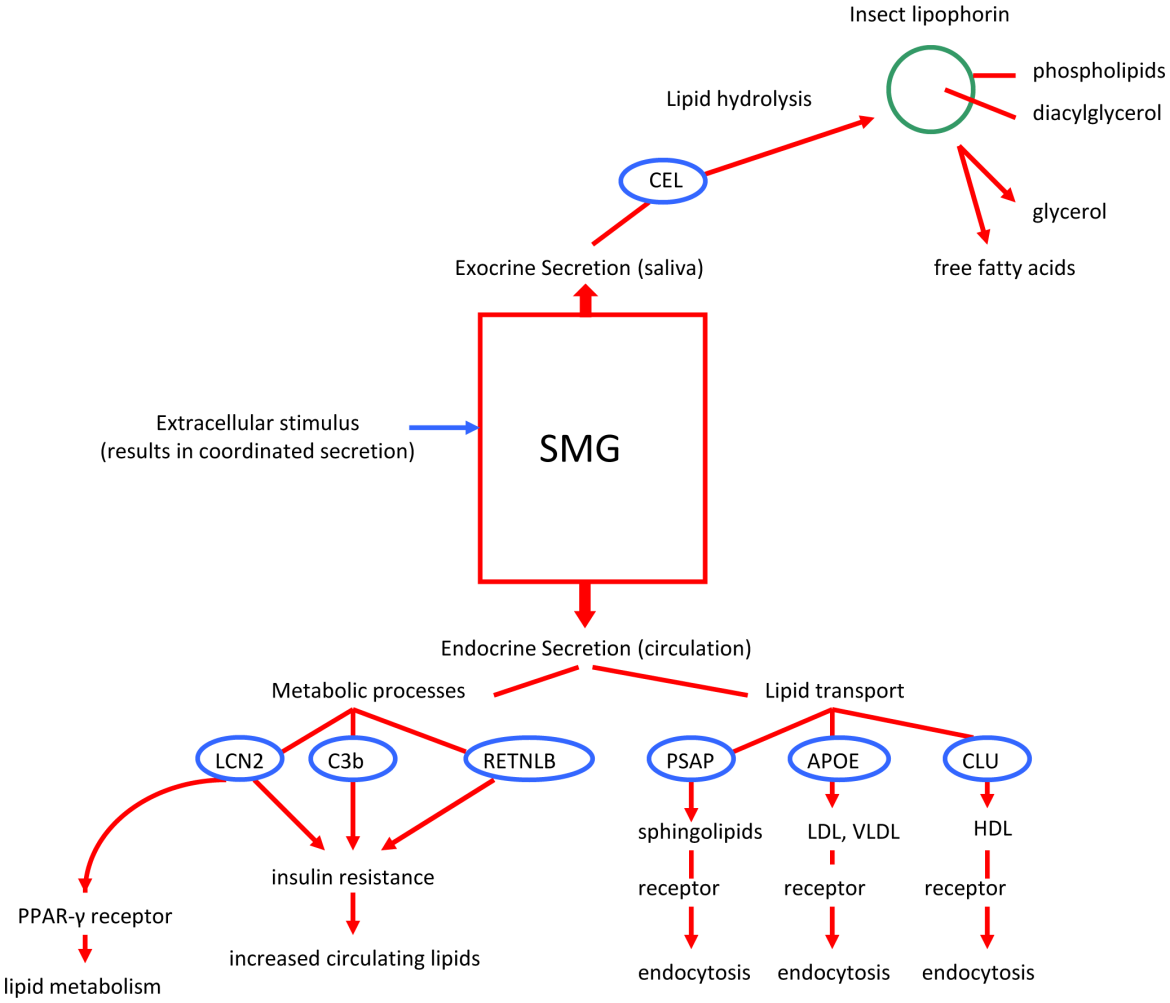
Diagram showing proposed adaptive roles of each of the genes recruited to expression in the secretory proteome of the *Myotis lucifugus* SMG.

The expression in *Myotis* SMG of seven genes whose encoded secretory products are associated with lipid metabolism is compatible with the hypothesis that this gland has an important adaptive role in processing exogenous insect lipids. It is logical to anticipate that salivary glands have roles in dietary adaptation, but in this instance its adaptive role apparently includes flight metabolism. Among the predicted SMG secretory proteins, one (CEL) hydrolyzes lipids, three (C3b, LCN2, RETNLB) are associated with insulin resistance, regulation of lipid metabolism, and fatty acids, and the remaining three (PSAP, APOE, CLU) all are associated with lipid transport and receptor-mediated endocytosis (Fig. 6). Insulin resistance is important because insulin promotes the clearance and storage of lipids while inhibiting their release into the circulation [83]. In fact, in addition to insulin resistance in particular, overproduction of any of these secretory proteins is associated with metabolic syndrome (type 2 diabetes) in humans [84], [85]. Coordinated SMG secretion of these proteins makes them quickly available in circulation. In *M. lucifugus*, adaptation has produced a metabolic strategy that also favors extremely rapid movement of lipids into the circulation followed by a period of sustained hyperlipidemia. It is clear that there are fundamental, but normal, metabolic differences between insectivorous bats, such as *M. lucifugus*, and humans.

We conclude that the seven genes expressed in *Myotis* SMG (Table 1; Fig. 6) compose an adaptive package as the encoded secretory proteins have interrelated—or possibly coordinated—functions. Orthologs to these seven genes are expressed in a variety of tissues in other mammals, but never linked together in a single organ or tissue. Co-expression of this set of genes in an organ where secretion is linked to feeding elucidates one evolutionary mechanism underlying adaptation. In effect, recruitment of genes ordinarily expressed and regulated in different tissues (ranging from pancreas and intestine to liver and adipose cells) and their subsequent expression in a single organ represents adaptation through creation of novel gene and protein networks. Dropping or adding proteins to an existing network, or creation of a novel network, has important adaptive potential [86]. Finally, it also is the case that salivary glands have both endocrine and exocrine secretory pathways and this adds significantly to their importance in adaptation [15], [87]. This conclusion about the adaptive role of salivary glands in mammals is strongly supported by elegant studies of mice that demonstrated a correlation between aggressive behavior and fighting and endocrine release of products including nerve growth factor from the SMG [88].

Among predicted secretory products, carboxyl ester lipase is a likely candidate for making exogenous lipids available quickly. In insects, most lipids are stored in the form of lipophorins, which consist primarily of diacylglycerol cores with phospholipid surfaces [21], [22]. The conventional carboxyl ester lipase (the enzyme in humans) has been shown to be especially effective with mono- and diacylglycerols and with phospholipids [56]. The human and rodent CEL isoforms retain their function when present in the stomach. If the *Myotis* SMG carboxyl ester lipase is released into saliva and if it also retains its functional capabilities in the stomach, it then is reasonable to hypothesize that at least the medium chain length insect lipids can be absorbed directly across the gastric mucosa, following the same pathway as some milk lipids in neonate humans and laboratory rodents [89]–[92]. In neonatal rats, medium chain lipids in milk pass through the stomach wall and can be detected in circulation within five minutes of feeding [89]. This rate approximates the rate at which insectivorous bats begin to use exogenous lipids to fuel their flight [13]. But milk and insects are obviously different physically. In order for rapid gastric absorption of lipids to occur in a flying insectivorous bat, the ingested prey must be finely comminuted before it reaches the stomach. This requirement is consistent with the conservative coronal morphology and complex occlusion patterns characteristic of molar dentition in insectivorous bats [5], [11], [93], [94]. The molar teeth have sharp, W-shaped shearing cusps that readily reduce prey to fine particles before swallowing.

Our study illustrates how an organ, the submandibular salivary gland, that at first blush has no obvious connection to the central feature of bat existence, namely, powered flight, plays a key and heretofore unexpected adaptive role in the evolution and maintenance of this unique ability among mammals.

## Methods and Materials

Our research was conducted in accordance with protocol number 26592 for wild animals approved prior to our study by the Institutional Animal Care and Use Committee (IACUC) of Pennsylvania State University. The IACUC follows NIH and USDA guidelines for animal research and every effort is made to minimize discomfort or pain and to use the minimal number of individuals. A male specimen of *Myotis lucifugus* was collected under scientific collecting permit #00098 (issued to Michael Gannon by the Pennsylvania Game Commission) at Shaver's Creek Environmental Center, Huntingdon County, Pennsylvania. This collection locality was chosen so that the SMG transcriptome would come from a bat within the same genetic lineage of *Myotis lucifugus* used for the Ensembl genome project. The bat was euthanized according to the approved protocol and then the right and left intact submandibular salivary glands were dissected in the field and immediately placed in liquid nitrogen. Tissue samples from this bat are archived in the Genetics Resources Collection at the Museum of Texas Tech University. In the laboratory, one entire gland weighting approximately 60 mg was homogenized in 1 ml of Trizol Reagent and total RNAs were isolated following manufacturer protocol (Life Technologies, Carlsbad, California, USA). RNA integrity was verified using a Bioanalyzer (Santa Clara, California, USA). The fragment library preparation was developed from Poly(A) selected RNAs and sequenced for one lane of SOLEXA GAIIx 75 base-pair paired end sequencing, yielding 29.3 million pairs of reads. Sequence data are available through GenBank Short Read Archive (accession number SRP031492).

The Ensembl *Myotis lucifugus* assembly and annotation Myoluc2.0.67 [95] was used for whole transcriptome analysis. A total of 29.3 million RNA-seq read pairs (58.6 m read fragments) were mapped to the assembly using Tophat v1.2.0 [96] (and Bowtie v0.12.7) with default parameters, and Rna-SeQC v1.1.7 [97] was used to obtain read mapping statistics. Table 3 provides statistics describing the mapped reads. ∼13 m read pairs and ∼39 m read fragments were mapped to the genome, with ∼93% of read pairs and ∼85% of read fragments mapping uniquely. ∼25% of reads mapped to known exons, and ∼47% mapped to intragenic regions. These statistics indicate that the sequencing data is robust and the read mapping was successful, especially given that the Myoluc genome is quite incomplete at more than 11,500 scaffolds. The mapped reads were assembled using Cufflinks version 1.0.3 with the Myoluc2.0.67 gene annotation as the reference annotation and otherwise using default parameters [98].

**Table 3:** Read mapping statistics.

Category	Value
Total Reads	∼2.9 M. read pairs/∼5.9 M. read fragments
Mapped Reads	∼1.3 M read pairs (45.8%)*/∼1.2 unique pairs (45.1% of total, 92.9% of mapped pairs)/∼3.9 total fragments (66.9%)*/∼3.3 uniquely mapped fragments (56.7% of total, 84.8% of mapped fragments)
Mapped Reads' Mismatch Rate	1.4%
Mapping to Gene Annotation	24.8% mapped to exons, 22.2% mapped to introns, 53% intergenic

From the assembled transcriptome we produced a putative secretory proteome for the *Myotis* SMG. Although we use the term ‘secretory proteome,’ we did not independently confirm the presence of any of the secretory proteins in SMG exocrine or endocrine secretions. For gene names in all cases we followed the HUGO Gene Nomenclature Committee's symbols and names.

To examine the possibility of adaptive amino acid replacements in the genes (or gene families) of functional interest detected in the transcriptomes, the *Myotis* loci identified in ENSEMBL were aligned with those from other mammal species in the same or sister clades in the ENSEMBL gene tree (s). In most instances this included orthologous genes in the megabat *Pteropus* using the ClustalW for codons routinely available in MEGA 5.05 [99]. Minor refinements of automated alignments were done manually. Decisions on which genes are included and on the corresponding alignments are critical to results of this analysis: to facilitate assessment or reanalysis, final alignments are available in Support Information 1. Analyses of episodic directional selection on codons then were carried out using the MEME routine implemented in http://www.datamonkey.org, using the general codon time-reversible model (REV, see 98) substitution model [100]. Sites were posited to be under directional selection at selected branches if they were: 1) significant at the P>0.05 level; and if they 2) also showed an Empirical Bayes Factor>20.

RepeatMasker web service was implemented to determine the overall occurrence of repetitive elements within genes of interest in the secretory proteome (RepeatMasker website. Available; http://www.repeatmasker.org/cgi-bin/WEBRepeatMasker, Accessed 2012 October 16). The complete nucleotide sequences for each gene were obtained from ENSEMBL and paralogs were aligned against the ‘ancestral’ sequence with ClustalW. These sequences were used as input with individualized introns and exons in the notation. The cross-match search engine and mammal DNA source option were implemented during analysis.
